# The autophagic degradation of Cav-1 contributes to PA-induced apoptosis and inflammation of astrocytes

**DOI:** 10.1038/s41419-018-0795-3

**Published:** 2018-07-10

**Authors:** Zi Chen, Sheng-Dan Nie, Min-Li Qu, Di Zhou, Liang-Yan Wu, Xia-Jie Shi, Ling-Ran Ma, Xin Li, Shan-Lei Zhou, Shan Wang, Jing Wu

**Affiliations:** 1 Department of Endocrinology, Xiang-Ya Hospital, Central South University, Changsha, China; 2 Department of Pharmaceutical Engineering, College of Chemistry and Chemical Engineering, Central South University, Changsha, China; 3 Institute of Clinical Medicine, Hunan Provincial People’s Hospital, The First Affiliated Hospital of Hunan Normal University, Changsha, China

## Abstract

The accumulation of palmitic acid (PA), implicated in obesity, can induce apoptotic cell death and inflammation of astrocytes. Caveolin-1 (Cav-1), an essential protein for astrocytes survival, can be degraded by autophagy, which is a double-edge sword that can either promote cell survival or cell death. The aim of this study was to delineate whether the autophagic degradation of Cav-1 is involved in PA-induced apoptosis and inflammation in hippocampal astrocytes. In this study we found that: (1) PA caused apoptotic death and inflammation by autophagic induction; (2) Cav-1 was degraded by PA-induced autophagy and PA induced autophagy in a Cav-1-independent manner; (3) the degradation of Cav-1 was responsible for PA-induced autophagy-dependent apoptotic cell death and inflammation; (4) chronic high-fat diet (HFD) induced Cav-1 degradation, apoptosis, autophagy, and inflammation in the hippocampal astrocytes of rats. Our results suggest that the autophagic degradation of Cav-1 contributes to PA-induced apoptosis and inflammation of astrocytes. Therefore, Cav-1 may be a potential therapeutic target for central nervous system injuries caused by PA accumulation.

## Introduction

In the central nervous system (CNS), lipids are majorly synthesized and metabolized by astrocytes^1,2^. Palmitic acid (PA), the most abundant saturated free fatty acid (FFA) in the diet, is elevated in plasma of the obese subjects^3,4^. Excessive PA could permeate the blood–brain barrier^5,6^, and then stimulate the production of proinflammatory cytokines and initiate caspase cascades, resulting in inflammation and apoptosis of astrocytes^7,8^. In order to provide an effective intervention for lipotoxicity of PA in CNS, further investigation is required to more precisely delineate the molecular mechanisms by which PA causes cellular damage to astrocytes.

Autophagy, a conserved evolutionary process in all eukaryotic cells, is responsible for the sequestration and delivery of damaged organelles and macromolecule into lysosomes for degradation, which acts as a pro-survival mechanism^9–12^. However, when autophagy is prolonged or overstimulated, it accelerates autophagic degradation of some survival-associated proteins^13–15^, leading to autophagic cell death, which seems like a hyper-stimulated self-eating of cells. It has been reported that autophagy aggravated the lipotoxicity of PA in endothelial cells^16^ and cardiomyocytes^17^, whereas reversed the damage caused by PA in human gastric cancer cells^18^ and adipocytes^19^, suggesting that the contribution of autophagy to cell fates, survival or death, varied in a cell type-dependent fashion. In the present study, we focus on the role of autophagy in PA-caused lipotoxicity in astrocytes.

Caveolin-1 (Cav-1), the principal structural component of caveolae membranes, which can modulate cell death and survival pathways via direct interaction with specific binding partners^20^, is expressed abundantly in astrocytes in CNS^21^. It has been reported that Cav-1 may influence fatty acid uptake by regulating surface availability of fatty acid translocase (FAT/CD36)^22^. Available evidence indicates that Cav-1 overexpression alleviates hypoxia-induced astrocyte apoptosis^23^, whereas Cav-1 knock-out (KO) enhances traumatic brain injury-induced neuroinflammation^24^. More importantly, Cav-1 can be degraded by hypoxia-induced autophagy in stromal fibroblasts^25^ and non-small-cell lung cancer cells^26^. However, whether the autophagic degradation of Cav-1 is involved in PA-induced lipotoxicity is not clear.

In this study, we have investigated whether or not the autophagic degradation of Cav-1 contributes to PA-induced apoptosis and inflammation in astrocytes, and confirmed the decline of Cav-1 in hippocampal astrocytes of high-fat diet (HFD) rats, in which the serum FFA is excessive. Our major findings are: (1) PA caused apoptotic death of astrocytes by autophagic induction; (2) PA increased the expression of proinflammatory cytokines and nuclear factor-κB (NF-κB) phosphorylation by autophagic induction; (3) Cav-1 was degraded by PA-induced autophagy and PA induced autophagy in a Cav-1-independent manner; (4) the overexpression of Cav-1 attenuated PA-induced apoptotic cell death and inflammation; (5) PA-induced apoptosis and inflammation could not be inhibited by autophagy inhibitor 3-methyladenine (3-MA) in Cav-1 silence cells; (6) chronic HFD induced apoptosis, inflammation, autophagy, and Cav-1 downregulation in the hippocampal astrocytes of rats.

## Results

### PA caused apoptotic death of astrocytes by autophagic induction

**Fig. 1:**
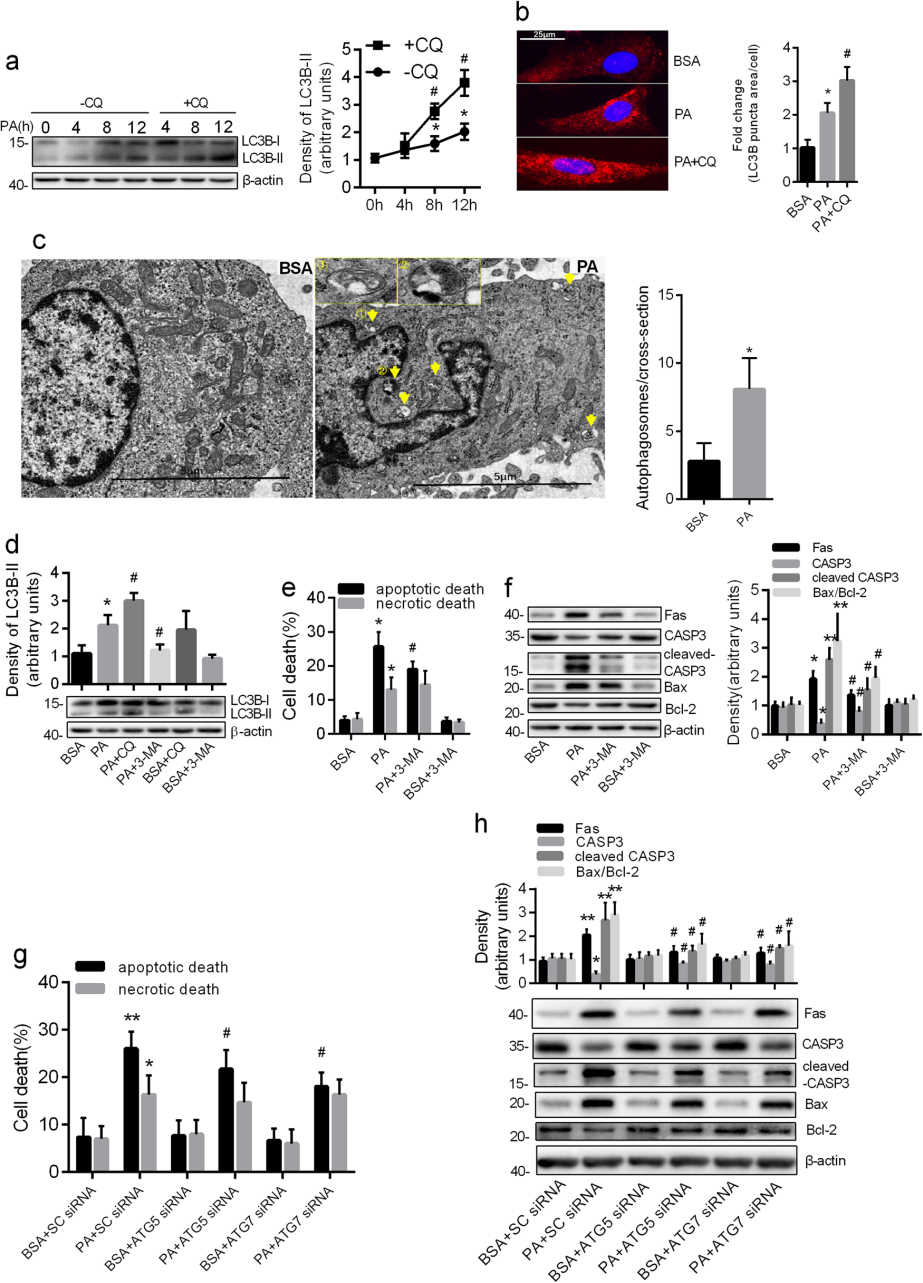
PA caused apoptotic death of astrocytes by autophagic induction. **a** Western blots showing the protein levels of LC3B-II in the astrocytes incubated with PA (0.25 mM) for the indicated times in the absence and presence of CQ (50 μM) (mean ± S.E.M. *n* = 3, *P < 0.05 vs. control; ^#^*P* < 0.05 vs. corresponding time without CQ). **b** Representative fluorescent images showing the LC3B puncta formation in astrocytes incubated with PA (0.25 mM, 12 h) in the absence and presence of CQ (50 μM). Scale bar, 25 μm. The LC3B puncta area per cell was analyzed by Image J software and the fold change in LC3B puncta area relative to the control was plotted (mean ± S.E.M. *n* = 3, *P < 0.05 vs. BSA; ^#^*P* < 0.05 vs. PA). **c** Representative transmission electron microscopy images of the astrocytes incubated with PA for 12 h. Yellow arrows denote autophagosomes. Scale bar, 5 μm. The number of autophagosomes per cross-sectioned cell were calculated (mean ± S.E.M. *n* = 10/group, *P < 0.05 vs. BSA). **d** Western blots showing the expression of LC3B-II in the astrocytes exposed to PA (0.25 mM, 12 h) in the absence and presence of 3-MA or CQ (mean ± S.E.M. *n* = 3, *P < 0.05 vs. BSA; ^#^*P* < 0.05 vs. PA). **e** The Annexin-V/PI labeling flow cytometry showing the apoptotic rate (Annexin V^+^/PI^−^ and Annexin V^+^/PI^+^) and necrotic rate (Annexin V^−^/PI^+^) of astrocytes exposed to 0.25 mM PA in the absence and presence of 3-MA (10 mM) (mean ± S.E.M. *n* = 3/group, *P < 0.05 vs. BSA; ^#^*P* < 0.05 vs. PA). **f** Western blots showing the expression of Fas, CASP3, cleaved CASP3, and Bax/Bcl-2 ratio in the astrocytes exposed to PA (0.25 mM, 12 h) in the absence and presence of 3-MA (10 mM) (mean ± S.E.M. *n* = 3, *P < 0.05, **P < 0.01 vs. BSA; ^#^*P* < 0.05 vs. PA). **g** The Annexin-V/PI assay showing the apoptotic rate (Annexin V^+^/PI^−^ and Annexin V^+^/PI^+^) and necrotic rate (Annexin V^−^/PI^+^) of ATG5 or ATG7-deficient cells incubated with PA (0.25 mM, 12 h) (mean ± S.E.M. *n* = 3/group, *P < 0.05, **P < 0.01 vs. BSA + SC-siRNA; ^#^*P* < 0.05 vs. PA + SC-siRNA). **h** Western blots showing the expression of Fas, CASP3, cleaved CASP3, and Bax/Bcl-2 ratio in ATG5 or ATG7-deficient cells incubated with PA (0.25 mM, 12 h) (mean ± S.E.M. *n* = 3, *P < 0.05, **P < 0.01 vs. BSA + SC-siRNA; ^#^*P* < 0.05 vs. PA + SC-siRNA)

Previous studies have demonstrated that autophagy can be induced by PA in several cell lines^16,19,27^. We determined whether PA-induced autophagy changed over a time course in purified cultured astrocytes verified by immunofluorescence (Supplementary Fig. 1). We measured the levels of LC3B-II protein accumulation (autophagic flux marker) in PA-treated astrocytes in the presence and absence of chloroquine (CQ, inhibitor of autophagic degradation). The results of western blots showed that the difference in the LC3B-II protein level in the presence of CQ compared to that in its absence, increased with prolonged incubation of cells (Fig. 1a), suggesting PA induced autophagic flux in a time-dependent manner. Consistently, increased LC3B after 12 h exposure to PA was observed by immunofluorescence (Fig. 1b). Furthermore, the formation of autophagic vesicle was detected by transmission electron microscopy. As shown in Fig. 1c, the autophagic vacuoles were observed in PA-treated astrocytes, but rarely seen in untreated cells.

To evaluate the functional role of autophagy in PA-induced cell death, we assessed apoptotic and necrotic death of PA-treated astrocytes by pretreating the cells with an autophagy inhibitor, 3-MA. We found that 3-MA treatment inhibited the increase of LC3B-II induced by PA, whereas CQ aggravated it (Fig. 1d). Interestingly, 3-MA could rescue PA-induced apoptotic death, but not necrotic death (Fig. 1e), accompanying the downregulation of the ratio of Bax/Bcl-2 and the expression of Fas and cleaved caspase 3 (CASP3) (Fig. 1f). Furthermore, when the autophagy genes ATG5 and ATG7 were knocked down in primary astrocytes (Supplementary Fig. 2a and b), both autophagic flux and apoptotic death induced by PA were inhibited (Supplementary Fig. 2c, Fig. 1g). Consistent with pharmacological blocking, gene-silencing of ATG5 or ATG7 also had no effect on PA-caused necrotic death (Fig. 1g). These results were evidenced by the detection of reduced Bax/Bcl-2 ratio and downregulated expression of Fas and cleaved CASP3 (Fig. 1h). These results suggest that autophagy activation is implicated in PA induced apoptotic death, but not necrotic death.

### PA increased the expression of proinflammatory cytokines and p-NF-κB by autophagic induction in astrocytes

**Fig. 2:**
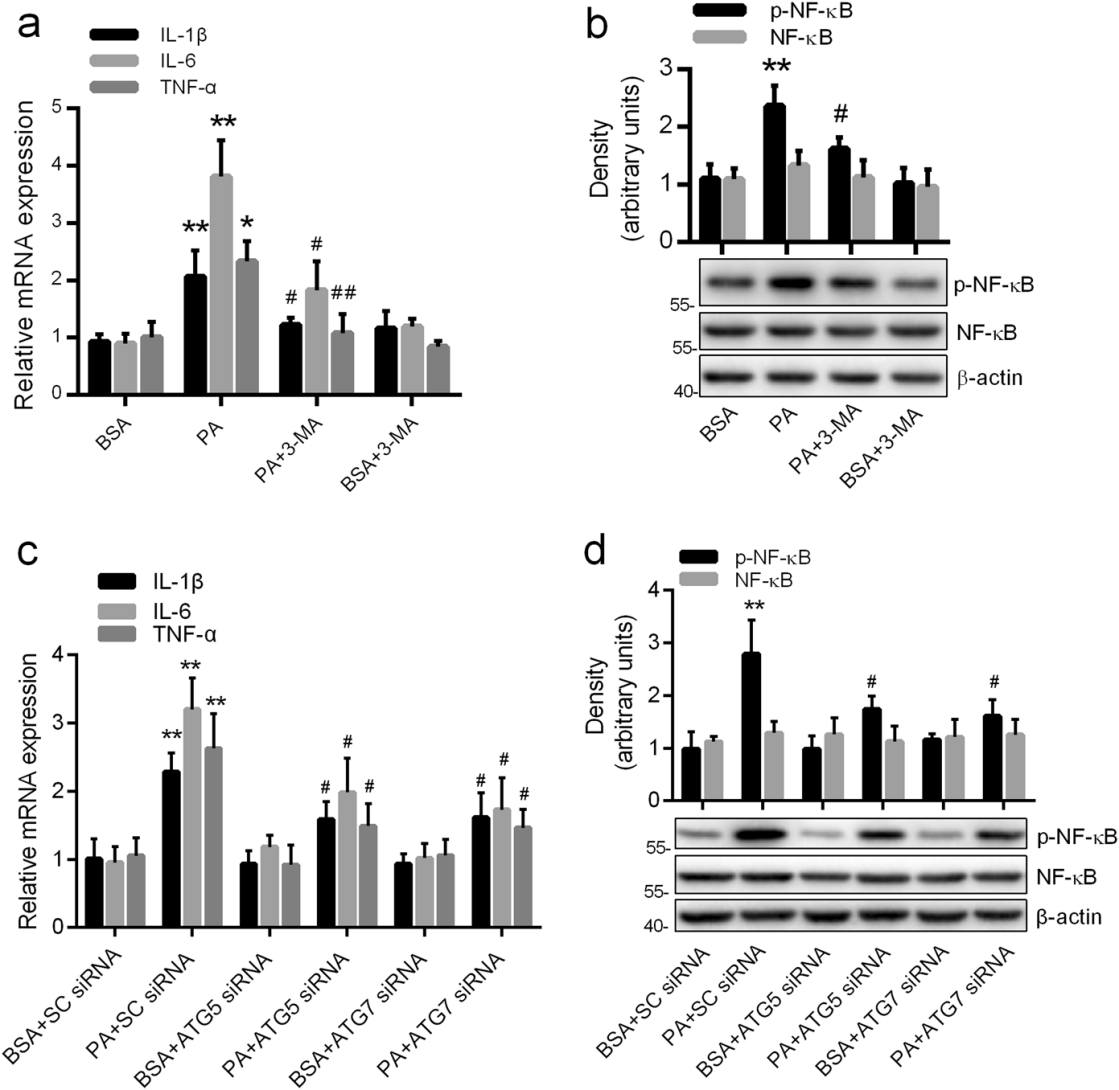
PA increased the expression of proinflammatory cytokines and p-NF-κB by autophagic induction in astrocytes. **a** Real-time qPCR and **b** western blots showing the mRNA levels of inflammatory cytokines (TNF-α, IL-1β, and IL-6) and the expression of p-NF-κB respectively in the astrocytes incubated with PA (0.25 mM, 12 h) in the absence and presence of 3-MA (10 mM) (mean ± S.E.M. *n* = 3, *P < 0.05, **P < 0.01 vs. BSA; ^#^*P* < 0.05, ^##^*P* < 0.01 vs. PA). **c** Real-time qPCR and **d** western blots showing the mRNA levels of inflammatory cytokines (TNF-α, IL-1β, and IL-6) and the expression of p-NF-κB, respectively, in ATG5 or ATG7 silence astrocytes exposed to PA (0.25 mM, 12 h) (mean ± S.E.M. *n* = 3, **P < 0.01 vs. BSA + SC-siRNA; ^#^*P* < 0.05 vs. PA + SC-siRNA)

As shown in Fig. 2a, the mRNA levels of interleukin-1β (IL-1β), interleukin-6 (IL-6), and tumor necrosis factor-α (TNF-α) were dramatically increased in the PA-treated group, compared with the control. The expression level of p-NF-κ B p65 at Serine-536 but not total NF-κB p65 showed a sharp and significant increase in the cells exposed to PA as compared to controls (Fig. 2b).

In order to investigate the contribution of autophagy to PA-induced inflammation, the blockage of autophagy by pharmacological or genetic means was used in our study. Compared with the PA-treated cells, the mRNA levels of IL-1β, IL-6, and TNF-α were significantly inhibited by 3-MA, ATG5 small interfering RNA (siRNA) or ATG7 siRNA (Fig. 2a, c). As predicted, the upregulation of p-NF-κB p65 at Serine-536 induced by PA was also reversed by the blockage of autophagy (Fig. 2b, d). These results indicate that autophagy activation is involved in PA-induced inflammation.

### Cav-1 was degraded by PA-induced autophagy

**Fig. 3:**
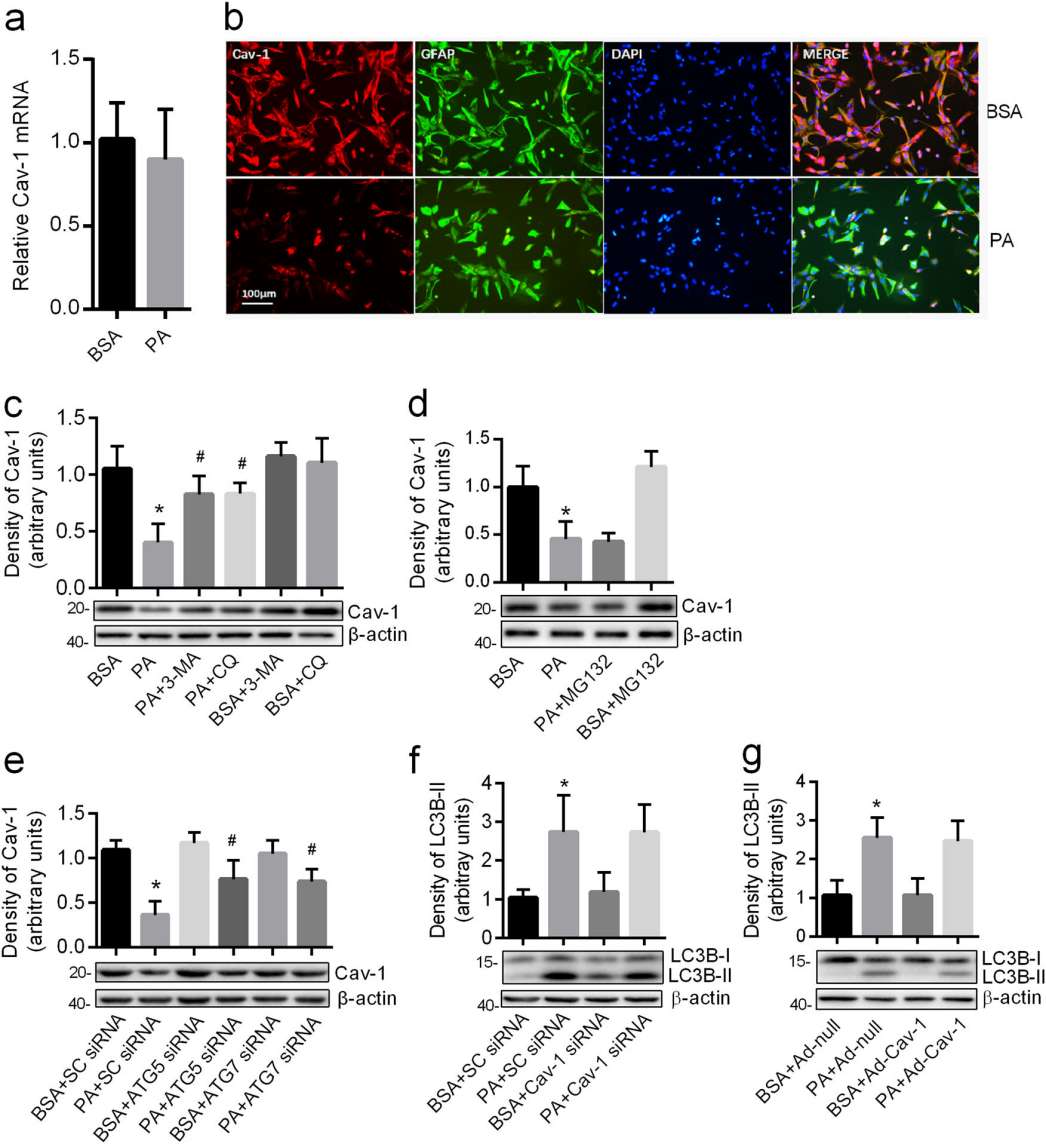
Cav-1 was degraded by PA-induced autophagy. **a** Real-time qPCR showing the expression of Cav-1 mRNA in the astrocyte exposed to PA (0.25 mM, 12 h) (mean ± S.E.M. *n* = 3). **b** The representative immunofluorescence images of Cav-1 in the astrocytes exposed to PA (0.25 mM, 12 h). Cav-1 was shown in red; GFAP was shown in green. Scale bar, 100 μm, *n* = 3. Western blots showing the expression of Cav-1 in the astrocytes incubated with PA (0.25 mM, 12 h) in the absence and presence of 3-MA (**c**) or CQ (**c**) or 10 μmol/L MG132 (**d**) (mean ± S.E.M. *n* = 3, *P < 0.05 vs. BSA; ^#^*P* < 0.05 vs. PA). **e** Western blots showing the expression of Cav-1 in ATG5 or ATG7-deficient astrocytes exposed to PA (0.25 mM, 12 h) (mean ± S.E.M. *n* = 3, *P < 0.05 vs. BSA + SC-siRNA; ^#^*P* < 0.05 vs. PA + SC-siRNA). **f** Western blots showing the expression of LC3B-II in Cav-1-deficient astrocytes exposed to PA (0.25 mM, 12 h) (mean ± S.E.M. *n* = 3, *P < 0.05 vs. BSA + SC-siRNA). **g** Western blots showing the expression of LC3B-II in Cav-1 overexpressing astrocytes exposed to PA (0.25 mM, 12 h) (mean ± S.E.M. *n* = 3, *P < 0.05 vs. BSA + Ad-null)

The mRNA expression of Cav-1 measured by real-time qPCR showed no statistical difference between PA-treated group and control group (Fig. 3a). However, the protein expression of Cav-1 by western blots demonstrated 54% reduction in the PA-treated group as compared with control (Fig. 3c). By employing immunofluorescence staining, we found that most of the Cav-1 signals were detected in the membrane and cytoplasm of cells, which was co-localized with the astrocytes marker glial fibrillary acidic protein (GFAP) (Fig. 3b). Consistent with our western blot analyses, decreased Cav-1 immunosignals were observed in the PA-treated group (Fig. 3b). These results indicate that PA decreases the expression of Cav-1 through a post-transcriptional mechanism. Pretreatment with the autophagy inhibitor 3-MA or lysosomal inhibitor CQ, but not with the proteasome inhibitor MG132, blocked the decline of Cav-1 protein induced by PA (Fig. 3c, d). Furthermore, the knockdown of either ATG5 or ATG7 rescued the level of Cav-1 caused by PA (Fig. 3e). Taken together, our results suggest that the loss of Cav-1 protein in PA-treated astrocytes is attributed to the increased autophagic degradation, but not proteasomal degradation.

Previous studies demonstrated that Cav-1 could regulate autophagy in several cells^28,29^. To explore the contribution of Cav-1 to autophagy in astrocytes, we transfected the astrocytes with Cav-1 siRNA and the knockdown of Cav-1 was confirmed by real-time qPCR and western blot (Supplementary Fig. 3a, b). As shown in Fig. 3f, the knockdown of Cav-1 could not increase the expression of LC3B-II independent of the presence of PA. In addition, we infected the astrocytes with adenovirus encoding Cav-1 and the overexpression of Cav-1 was confirmed by real-time qPCR and western blot (Supplementary Fig. 3c, d). No significant difference of LC3B-II expression was detected between Cav-1 overexpressing cells and the control cells in the presence or absence of PA (Fig. 3g). These data indicated that PA induced autophagy in a Cav-1-independent manner.

### The degradation of Cav-1 was responsible for PA-induced autophagy-dependent apoptotic cell death and inflammation

**Fig. 4:**
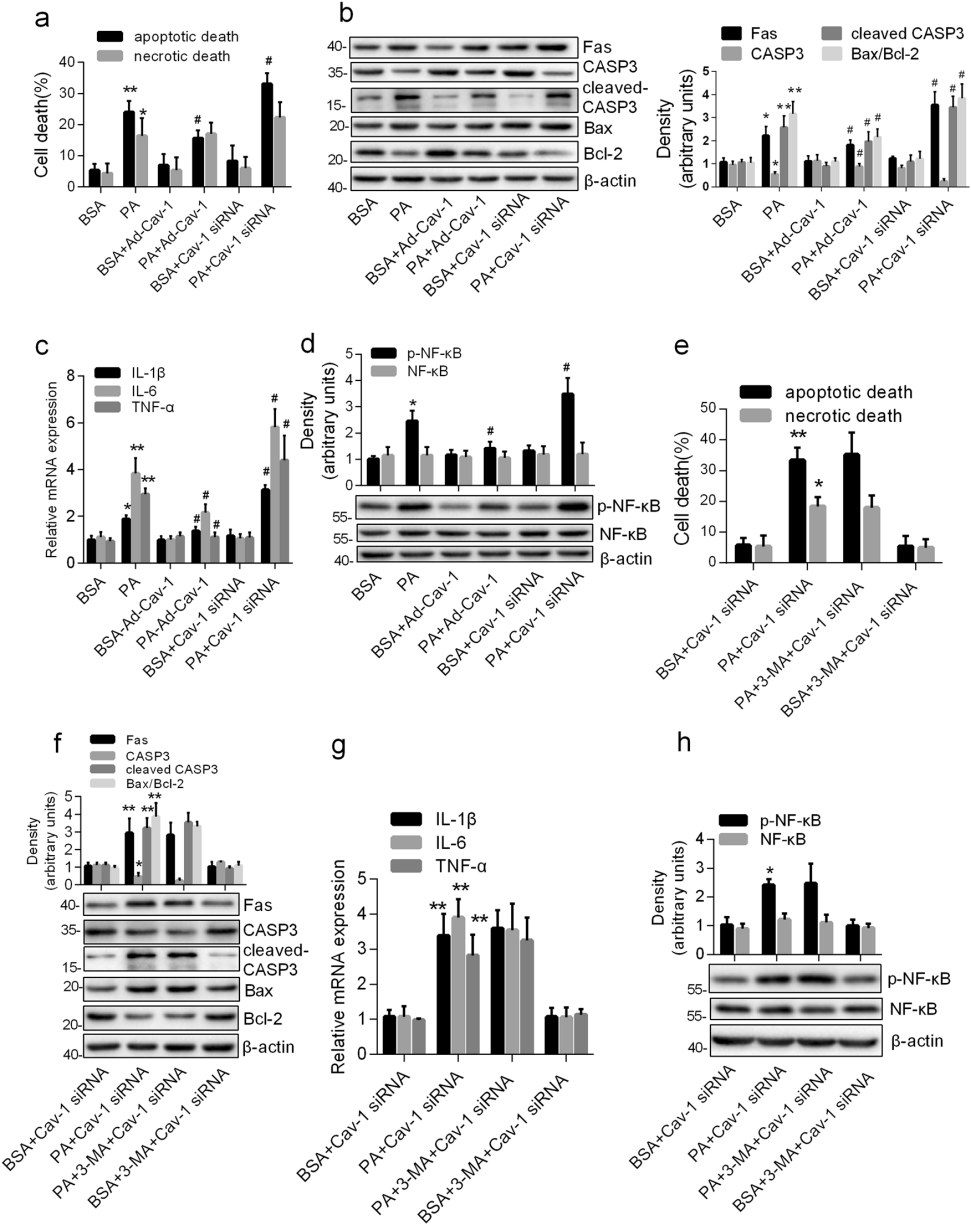
The degradation of Cav-1 was responsible for PA-induced autophagy-dependent apoptotic cell death and inflammation. The astrocytes with Cav-1 knockdown or Cav-1 overexpression were incubated with PA (0.25 mM) for 12 h (**a**–**d**). **a** Annexin-V/PI labeling flow cytometry showing the percentages of apoptotic cells (Annexin V^+^/PI^−^ and Annexin V^+^/PI^+^) and necrotic cells (Annexin V^−^/PI^+^) (mean ± S.E.M. *n* = 3/group, *P < 0.05, **P < 0.01 vs. BSA; ^#^*P* < 0.05 vs. PA). **b** Western blots showing the expression of Fas, CASP3, cleaved CASP3, and Bax/Bcl-2 ratio. **c** Real-time qPCR showing the relative mRNA levels of TNF-α, IL-1β, and IL-6. **d** Western blots showing the p-NF-κB p65 and total NF-κB p65 (mean ± S.E.M. *n* = 3, *P < 0.05, **P < 0.01 vs. BSA; ^#^*P* < 0.05 vs. PA). Cav-1-deficient astrocytes were exposed to PA (0.25 mM, 12 h) in the absence and presence of 3-MA (**e**–**g**). **e** Annexin-V/PI labeling flow cytometry showing the percentages of apoptotic cells (Annexin V^+^/PI^−^ and Annexin V^+^/PI^+^) and necrotic cells (Annexin V^−^/PI^+^) (mean ± S.E.M. *n* = 3/group, *P < 0.05 vs. BSA + Cav-1-siRNA). **f** Western blots showing the expression of Fas, CASP3, cleaved CASP3, and Bax/Bcl-2 ratio. **g** Real-time qPCR showing the relative mRNA levels of TNF-α, IL-1β, and IL-6. **h** Western blots showing the p-NF-κB p65 and total NF-κB p65 (mean ± S.E.M. *n* = 3, *P < 0.05, **P < 0.01 vs. BSA + Cav-1-siRNA)

To delineate the role of Cav-1 in PA-induced cell death and inflammation, the astrocytes with or without transient Cav-1 overexpression or knockdown were exposed to PA. As shown in Fig. 4a, overexpression of Cav-1 attenuated PA-caused apoptotic death, whereas downregulation of Cav-1 dramatically worsened it, with no effect on necrotic death. Consistently, Cav-1 overexpression inhibited PA-induced increase in Bax/Bcl-2 ratio, Fas and cleaved CASP3, whereas Cav-1 silence aggravated them (Fig. 4b). In addition, the mRNA expression of TNF-α, IL-1β, and IL-6, and the protein expression of p-NF-κB but not total NF-κB were inhibited by Cav-1 overexpression, but worsened by Cav-1 knockdown in PA-treated astrocytes (Fig. 4c, d). Collectively, our results indicate that the degradation of Cav-1 contributes to PA-induced cell apoptosis and inflammation in astrocytes.

To explore whether Cav-1 degradation is responsible for PA-induced autophagy-dependent apoptosis and inflammation, we observed the role of 3-MA in PA in Cav-1 silenced cells. As shown in Fig. 4e, the inhibition of PA-induced apoptotic death by 3-MA disappeared in Cav-1 knockdowned cells. Consistently, the downregulation of anti-apoptotic protein and upregulation of proapoptotic protein induced by PA could not been inhibited by 3-MA in Cav-1 silence cells (Fig. 4f). Also, the downregulation of proinflammatory cytokines and p-NF-κB by 3-MA was also abolished by Cav-1 knockdown in PA-treated astrocytes (Fig. 4g, h). Apparently, these results suggest that Cav-1 degradation contributes to PA-induced autophagy-dependent apoptosis and inflammation in astrocytes.

### Chronic HFD induced Cav-1 degradation, apoptosis, autophagy, and inflammation in the hippocampal astrocytes of rats

**Fig. 5:**
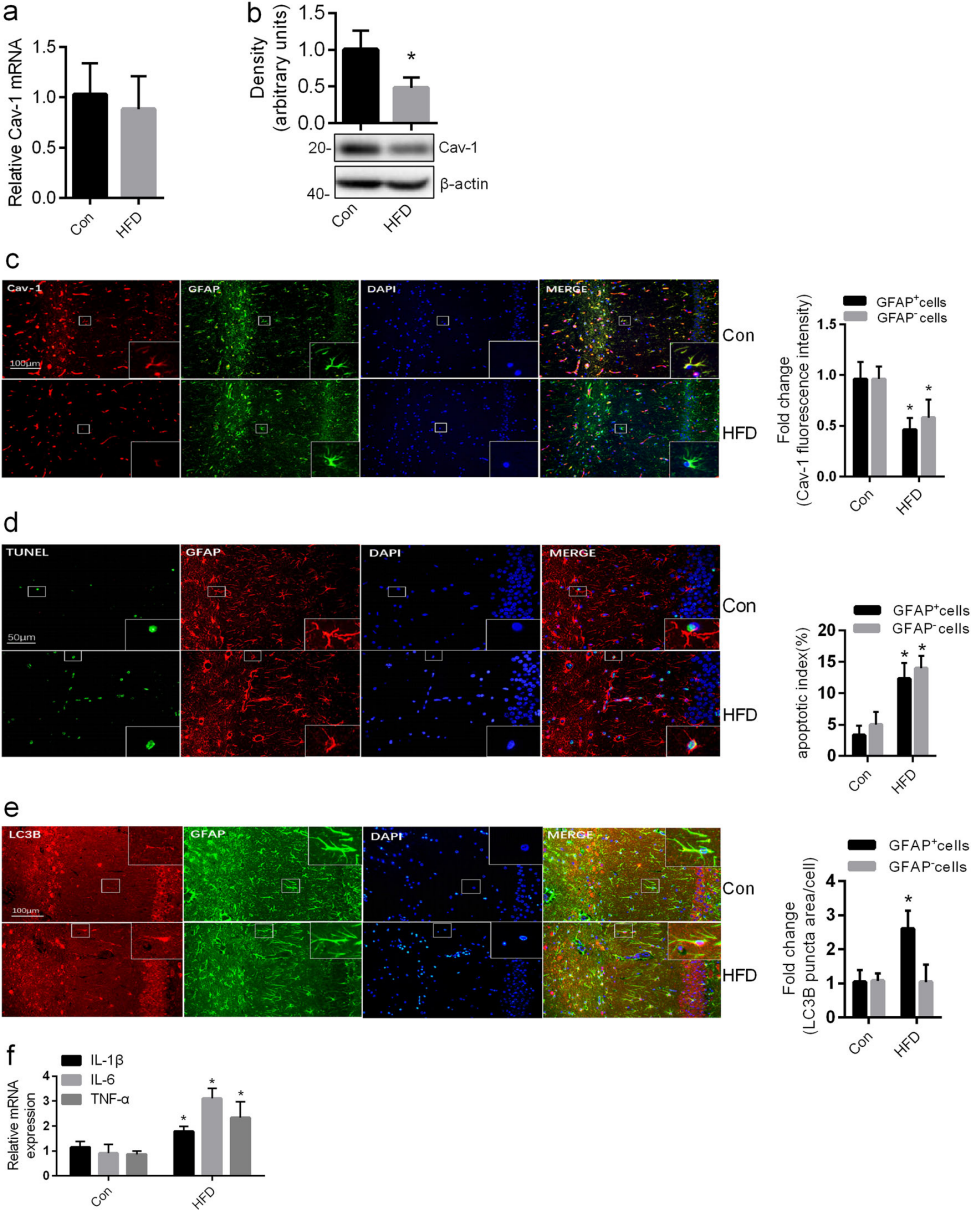
Chronic HFD induced Cav-1 degradation, apoptosis, autophagy, and inflammation in the hippocampal astrocytes of rats. The HFD group rats were exposed to high-fat diet for 20 weeks. Con normal control rats, HFD high-fat diet rats. **a** Real-time qPCR and **b** western blots showing the expression of Cav-1 in the hippocampi of control and HFD rats. **c** Representative images of the Con group and the HFD group were immunostained by anti-Cav-1. Cav-1 was shown in red; GFAP was shown in green. Scale bar, 100 μm. The Cav-1 fluorescence intensity of GFAP^+^ cells and GFAP^−^ cells in the hippocampi of control and HFD rats were analyzed by the Image J software and the fold change in Cav-1 fluorescence intensity relative to the control was plotted. **d** Representative images of the Con group and the HFD group were immunostained with GFAP and TUNEL. TUNEL was shown in green; GFAP was shown in red. Scale bar, 50 μm. The percentage of TUNEL^+^ GFAP^+^ cells and TUNEL^+^ GFAP^−^ cells in the hippocampi of control and HFD rats were calculated by the Image-Pro Plus software. **e** Representative images of the Con group and the HFD group were immunostained with GFAP, DAPI, and LC3B. LC3B was shown in red; GFAP was shown in green. Scale bar, 100 μm. The LC3B puncta area of GFAP^+^ cells and GFAP^−^cells in the hippocampi of control and HFD rats were analyzed by the Image J software and the fold change in LC3B puncta area relative to the control was plotted. **f** Real-time qPCR showing the relative levels of TNF-α mRNA, IL-1β mRNA, and IL-6 mRNA of the hippocampi of control and HFD rats (mean ± S.E.M. *n* = 5/group, *P < 0.05 vs. Con)

Having confirmed the action of PA on astrocytes in vitro experiments, we next sought to examine whether excessive FFA is associated with the decline of Cav-1 in vivo. To answer this question, we detected the level of Cav-1 in the hippocampi of chronic HFD rats, in which the serum concentrations of TG and FFA were significantly increased (Supplementary Tab. 2). As observed in the vitro model, the protein levels but not the mRNA levels of Cav-1 was decreased in the hippocampi of HFD rats as compared with that in the controls (Fig. 5a, b). Furthermore, we found that weak immunoreactivities of Cav-1 were observed in hippocampal astrocytes in the chronic HFD brains, as compared to the control (Fig. 5c). Associated with the downregulation of Cav-1 expression, upregulated ratio of terminal deoxynucleotidyl transferase dUTP nick end labeling (TUNEL)-positive astrocytes and increased LC3B puncta area of astrocytes were observed in the hippocampus of HFD rats (Fig. 5d, e). And, the mRNA expression levels of proinflammatory cytokines (IL-1β, TNF-α, and IL-6) of the hippocampi were significantly increased in HFD rats compared with control rats (Fig. 5f).

## Discussion

Several studies focus on PA-induced cell death, including apoptosis^30^ and necrosis^31^. Zhen Wang et al.^8^ found that the exposure of cortical astrocytes to PA (0.2 mM for 24 h) caused largely apoptosis, but little necrosis. In our study, the incubation of hippocampal astrocytes with PA (0.25 mM for 12 h) caused not only apoptosis but also significant necrosis. It has been demonstrated that rat hippocampal astrocytes are more sensitive to oxygen-glucose deprivation (OGD) than cortical astrocytes^32^, suggesting that sensitivity of astrocytes on harmful stimuli might be dependent on the heterogeneity of astrocytes in different regions^33^.

Appropriate autophagy degrades damaged organelles and molecules and provides materials to support metabolism, protecting cells from insult^34^. However, when the level of autophagy rises to certain extent, some functional proteins such as Cav-1 ^26^, catalase^13^, and dBruce^14^ were degraded, leading to apoptosis^26^, which could be inhibited by preventing the destructive degradation of these functional proteins^15^. Previous studies reported several molecular mechanisms involved in PA-induced autophagy in different cell models, such as the Ca^2+^ leakage of the endoplasmic reticulum (ER) in endothelial cells^16^, the activation of transcription factor p8-p53-PKC α signaling in human gastric cancer MKN45 cells^18^, the modulation of protein kinase C in MEFs cells^35^, the promotion of JNK2 signaling pathway in hepatocytes^36^ and the dysfunction of mitochondria and excess of reactive oxygen species (ROS) in podocytes^37^ and hepatocytes^38^. The autophagy plays a protective role against PA-induced damage in human gastric cancer MKN45 cells^18^ and 3T3-L1 adipocytes^19^, but a destructive role in endothelial cells^16^, suggesting that the role of autophagy induced by PA maybe cell-type-dependent. In the current study, we demonstrated for the first time that PA caused apoptotic death of astrocytes by autophagic induction. We speculated that the treatment of PA (0.25 mM for 12 h) might cause a high level of destructive autophagy in hippocampal astrocytes, which are more sensitive to insult. Notably, autophagy inhibitor 3-MA inhibited but not totally block PA-induced apoptosis (Fig. 1e), indicating that other signaling pathway might be involved in the mechanism of apoptosis induced by PA. As reported in previous studies, PA could induce apoptosis by ER stress or oxidative stress^39^.

In our present study, we also demonstrated that PA increased the expression of proinflammatory cytokines and p-NF-κB by autophagic induction. Similar to PA, cocaine could upregulate the expression of proinflammatory cytokines through autophagic induction in astrocytes^40^. The autophagic degradation of the NF-κB inhibitor IkBα is a vital pathway to activate NF-κB and increase NF-κB nuclear translocation and transcription activity^41^. Recent evidence suggests that autophagy-induced production of proinflammatory cytokines such as IL-1β, TNF-α, and IL-6 via NF-κB activation in H5N1 pseudotyped viral particle (H5N1pps)-treated A549 cells^42^. Further study is required to investigate whether the autophagic degradation of IkBα or other anti-inflammation-related proteins contributes to the proinflammatory response induced by PA.

Cav-1, a key protein abundant in astrocytes^21^, was implicated in several pathological processes, including apoptosis^23^, inflammation^43^, and autophagy^26^. Our study showed that PA treatment significantly reduced the protein levels of Cav-1 but not the mRNA levels in cultured hippocampal astrocytes. Also, we found that weak immunoreactivities of Cav-1 were observed in the astrocytes of the hippocampus in the chronic HFD brains, as compared to the control. Similarly, the downregulation of Cav-1 was also reported in both thoracic aorta endothelium^44^ and liver of HFD mice^45^. It has been reported that Cav-1 co-localized with several autophagy proteins such as ATG5, ATG12, and LC3B in autophagosome in endothelial cells^46,47^. The autophagic degradation of Cav-1 has been demonstrated in several cell models: hypoxia-induced autophagic degradation of Cav-1 in A549^26^; breast cancer cells-induced autophagic degradation of Cav-1 in immortalized fibroblasts^48^. Therefore, we investigated whether the downregulation of Cav-1 was due to PA-induced autophagy. In our present study, we demonstrated that the loss of Cav-1 protein was attributed to PA-induced autophagic degradation, but not proteasomal degradation. Although the regulation of autophagy by Cav-1 has been reported in several cell models^46,49^, we showed that neither Cav-1 overexpression nor Cav-1 knockdown does affect the autophagy process in PA-treated hippocampal astrocytes. Recently, it has been reported that, in primary astrocytes derived from prnp^+/+^ or prnp^0/0^ mice, suppressed autophagy was due to the higher levels of Cav-1 in lipid raft, but not the total levels of Cav-1^50^. Further study is required to elucidate the role of Cav-1, especially the cav-1 distribution, in autophagy in astrocytes.

Cav-1 is essential for cellular survival, and its overexpression alleviated ischemic death of cortical neurons^51^ and hypoxia-mediated apoptosis of astrocytes^23^. In our present study, we found that the overexpression of Cav-1 attenuated PA-caused apoptotic death, whereas the downregulation of Cav-1 dramatically worsened it, with no effect on necrotic death. More importantly, our results showed that the inhibition of PA-induced apoptotic death by 3-MA disappeared in Cav-1 knockdowned cells, suggesting that the degradation of Cav-1 is responsible for PA-induced autophagy-dependent apoptotic cell death. Although the anti-apoptotic role of Cav-1 has also been demonstrated in cortical astrocytes exposed to OGD^23^, the mechanisms by which Cav-1 alleviates apoptosis remain unclear. Our study showed that the overexpression of Cav-1 inhibited the upregulation of Fas induced by PA, suggesting Fas maybe implicated in the anti-apoptotic role of Cav-1. Consistent with our results, the Fas expression was increased in Cav-1^−^/^−^ lung fibroblasts^47^. Again, other signaling pathways were reported to be involved in the cytoprotective role of Cav-1: Cav-1 protected against hypoxia-induced astrocyte apoptosis via the Ras/Raf/ERK pathway^23^; Cav-1 prevented the ischemic cell death of neurons via Src kinase and ERK 1/2 pathway^51^; Cav-1 abrogated TGF-β triggered apoptosis through non-Smad AKT signaling in hepatocytes^52^; Cav-1 protected against hepatic ischemia/reperfusion injury by ameliorating peroxynitrite-induced cellular damage^53^.

In addition to the anti-apoptotic properties of Cav-1, its role in anti-inflammation was evaluated in our present study. Our results indicated that the autophagic degradation of Cav-1 contributes to PA-induced inflammation through NF-κB activation. The inhibition of inflammation by Cav-1 has been demonstrated in different cells and animal models: Cav-1 overexpression decreased LPS-induced inflammation in macrophages^54^; Cav-1 scaffolding domain peptide inhibited psoriasis-like skin inflammation induced by imiquimod^55^; Cav-1 deletion aggravated brain inflammation induced by traumatic brain injury^24^ and lung inflammatory response to LPS^56^. Yet, paradoxically, Cav-1 Tyr14 phosphorylation mediated inflammation through interacting with TLR4 ^57–59^, suggesting the phosphorylation status of Cav-1 protein influences the functional role of Cav-1 in inflammation.

Epidemiological studies indicate that a high-fat dietary contributes to neurodegenerative disease^60,61^. The current study suggests that PA or HFD may accelerate neurodegenerative diseases through arrest of neuron cell cycle in the G2/M phase and elevation of endoplasmic reticular stress^60^. Consider for the damage of astrocytes also contributes to the development of neurodegenerative diseases, studying how PA induced cellular damage of astrocytes might reveal the novel mechanism by which PA/HFD aggravates neurodegenerative diseases.

Together, our results demonstrate the autophagic degradation of Cav-1 may be responsible for PA-induced apoptosis and inflammation in hippocampal astrocytes (Supplementary Fig. 5). Therefore, strategies to inhibit the autophagic degradation of Cav-1 may prove to be beneficial to reverse lipotoxicity caused by PA accumulation in CNS.

## Materials and methods

### Antibodies and reagents

Antibodies and their sources: antibodies against LC3B (2775), CASP3/caspase3 (9662), cleaved CASP3/caspase3 (9661), GFAP (3670), Bcl-2 (2870), and Bax (2772) were obtained from Cell Signaling Technology. Antibodies against ATG5 (ab108327), ATG7 (ab52472), GFAP (ab134436) and Fas (ab82419), NF-κB p65 (ab16502), Phospho-NF-κB p65 (Ser536) (ab86299) were purchased from Abcam. Anti-Cav-1(sc-894) for western blot analysis and immunofluorescence was obtained from Santa Cruz Biotechnology. Anti-β-actin (A1978) was obtained from Sigma. PA (C16:0, saturated fatty acid), CQ, and 3-MA were from Sigma. MG132 was from selleckchem (USA). The adenoviral Cav-1 (Ad-Cav-1) was kindly provided by Dr. Duan-Fang Liao (Hunan University of Traditional Chinese Medicine, Hunan, China).

### PA treatment, transfection, and infection in cultured hippocampal astrocytes

Hippocampal astrocytes were prepared from 1-day-old male Sprague-Dawley (SD) rat pups as described by Cristóvão Albuquerque et al.^62^. Briefly, after 0.125% Trypsin-EDTA (Gibco Life Technologies, USA) digestion, the hippocampal cells surgically isolated were seeded on 25 cm^2^ flasks in Dulbecco’s modified Eagle's medium (DMEM) (Gibco Life Technologies, USA) containing 10% fetal bovine serum and 1% (v/v) antibiotics mixture (penicillin/streptomycin) in a humidified incubator with 5% CO_2_ at 37 °C. To remove weakly adherent microglia and oligodendrocytes, flasks were orbitally shaken overnight (200 rpm at 37 °C) when the culture reached confluence. The astrocytes were passaged three times for further purification before use. Using this method, cultures containing >95% astrocytes were obtained, which was verified by immunostaining with GFAP (ab134436) (Supplementary Fig. 1). The experiments were carried out as following:

(1) PA treatment: PA (0.25 mΜ) was complexed with bovine serum albumin (BSA, fatty acid-free, low endotoxin) (Millipore, Billerica, MA, USA) at a molar ratio of 3:1 (fatty acid/albumin) in serum-free DMEM. The astrocytes were incubated with serum-free DMEM overnight and then treated with freshly prepared PA or BSA for 4, 8, or 12 h.

(2) Viral infection: The astrocytes were infected using either Ad-Cav-1 or Ad-null as previously described^63^. The efficiency of infections was determined by real-time quantitative PCR (qPCR) and western blot. The infected astrocytes were incubated with PA or BSA after 48 h infection.

(3) Transfection: At 70–80% confluence, the astrocytes were transfected with Cav-1 siRNA supplied by Invitrogen or transfected with ATG5 siRNA or ATG7 siRNA obtained from RiboBio as per the manufacturer’s instructions, respectively. The efficiencies of the siRNA were confirmed by real-time qPCR and western blot. The transfected astrocytes were exposed to PA for 12 h after 48 h transfection.

(4) CQ and 3-MA treatment: the transfected and non-transfected astrocytes were pretreated with CQ (50 μM) or 3-MA (10 mM) for 1 h prior to the exposure of PA for the indicated times.

### Real-time qPCR

Total RNA isolated by TRIzol reagent (Invitrogen, USA) was converted to complementary DNA (cDNA) by a cDNA Synthesis Kit (Gene Copoeia, USA) as per the manufacturer’s instructions. Subsequently, the cDNAs were amplified by real-time qPCR with specific primers (Supplementary Tab. 1) and SYBR-Green dye (Gene Copoeia, USA). PCRs were performed with the following parameters: pre-denaturation at 95 °C for 5 min, 40 cycles of denaturation at 95 °C for 10 s, annealing at 60 °C for 20 s, extension at 72 °C for 30 s. β-Actin was used as an internal control.

### Western blot analysis

Having experiments performed, the hippocampi were dissected from rat brains after perfusion with saline and sacrificed under deep anesthesia. The protein of hippocampi was homogenized and extracted as described previously^64^. Briefly, samples were homogenized in iced RIPA Lysis Buffer (Beyotime, China) containing 1 mM PMSF and 0.1% cocktail (Sigma, USA). Protein concentration was determined using a BCA Protein Assay Kit (Beyotime, China).

Astrocytes were washed with cold phosphate-buffered saline and lysed with RIPA Lysis Buffer (Beyotime, China) containing 1 mM PMSF and 0.1% cocktail (Sigma, USA) on ice. The cell extracts were obtained via centrifuging (13,000 × *g*, 20 min) at 4 °C. Twenty micrograms protein samples were separated on SDS-polyacrylamide gels (SDS-PAGE). The separated proteins were transferred onto PVDF membranes (Millipore, USA) which were incubated with the primary antibodies after the block of non-specific protein binding sites using 5% non-fat milk. Membranes were then washed and reacted with secondary antibodies (ZSGB-BIO, China). Each protein band was quantified by the Image J analysis system (NIH, Bethesda, MD, USA) and densitometrically normalized to that of β-actin. Again, we analyzed the Bax/Bcl-2 ratio rather than Bax and Bcl-2 alone, because the Bax/Bcl-2 ratio appears more important in determining the apoptotic potential of cells than the expression of Bax or Bcl-2 individually^65^.

### Immunofluorescence

For immunofluorescence, the whole brain was dissected and fixed in 4% paraformaldehyde prior to the embedment in paraffin. The brains were sectioned coronally at 5-μm thickness and 1% bovine serum albumin (BSA) (Sigma, USA) was used to avoid the non-specific staining on the sections. The sections were incubated with GFAP (ab134436) and Cav-1 or LC3B prior to exposure to Alexa Fluor 594-conjugated goat anti-rabbit secondary antibody (ZSGB-BIO, China) and Alexa Fluor 488-conjugated donkey anti-chicken secondary antibody (ZSGB-BIO, China). The slides were stained with DAPI and mounted.

The astrocytes were plated onto 20 mm diameter glass coverslips and starved overnight by serum-free DMEM before the PA treatment with or without CQ for 12 h. The astrocytes were harvested and fixed in 4% paraformaldehyde. The fixed astrocytes were permeabilized by 0.3% Triton-X-100 and subsequently incubated with 1% BSA. The astrocytes were labeled with primary antibodies GFAP (ab134436) and Cav-1 or LC3B prior to incubation with Alexa Fluor 594-conjugated goat anti-rabbit secondary antibody (ZSGB-BIO, China) or Alexa Fluor 488-conjugated donkey anti-chicken secondary antibody (ZSGB-BIO, China). The nucleus was stained with DAPI before the slides were mounted. The fluorescence images were randomly taken by a wide-field fluorescence microscope (Leica, Wetzlar, Germany) at 200× magnification. Fields of view were randomly chosen by an observer blinded to experimental groups and measured with Image J software.

### Annexin V-FITC/PI staining

The astrocytes were collected and stained using Annexin V-FITC and propidium iodide (PI) staining kit obtained from BD Biosciences as per the protocol provided by the manufacturer. Briefly, the astrocytes were resuspended and incubated with Annexin V-FITC and PI for 15 min. The dead cells were detected within 1 h using flow cytometry purchased from Becton Dickinson.

### Transmission electron microscopy

The cells were harvested and fixed in a solution containing 2.5% glutaraldehyde in 0.1 M sodium cacodylate overnight and then samples were processed in the Electron Microscopy Core at Xiang-Ya Hospital, Central South University. Cell pellets were postfixed with 1% osmium tetroxide and dehydrated. The samples were embedded before being cut into ultrathin sections. The sections were examined on a Hitachi HT7700 electron microscope (Hitachi, Tokyo, Japan) after uranyl acetate and lead nitrate staining.

### Animal preparations

The 2-month-old male SD rats were supplied by the Experimental Animal Center of Central South University (Hunan, China) and housed two per cage under a 12 h light/dark cycle. After acclimation with ad libitum access to a standard chow diet and sterile water for 1 week, SD rats were randomly divided into two groups: one group (*n* = 5) continuing with standard rat chow (17% kcal from fat) and the other group (*n* = 5) changing to the HFD (60.3% kcal from fat, purchased from SLAC Laboratory Animal Co. Ltd, Hunan, China) for 20 weeks^66^ for immune fluorescence. Additional control rats (*n* = 5) and HFD rats (*n* = 5) prepared as described above were for real-time PCR and western blot. The procedures above were conducted according to the guidelines of the NIH Guidelines for Care and Use of Laboratory Animals.

### Biochemical measurements

Serum triglyceride (TG), total cholesterol (TC), and FFA levels were measured after 20 weeks of dietary interventions. All the animals were fasted for 12 h before blood collection by cardiac puncture with the animals under deep anesthesia. Serum cholesterol and triglyceride were evaluated by commercial kits bought from Beijing Applygen Technologies Inc. The levels of FFAs was quantified using a kit from Wako Pure Chemicals (Richmond, VA, USA).

### TUNEL staining assay

The 5 μm paraffin brain sections were prepared as previously described. A commercial kit (In Situ Cell Death Detection Kit; Roche, Penzberg, Germany) was used for TUNEL labeling according to the manufacturer’s instructions. In brief, sections were incubated with the reaction mixture containing terminal deoxynucleotidyl transferase (TdT) and fluorescein-conjugated deoxyuridine triphosphate (dUTP) for 1 h at 37 °C. After TUNEL staining, sections were incubated with antibody for GFAP (3670) to identify astrocyte. Then sections were exposed to secondary antibody Alexa Fluor®594 anti-mouse Immunoglobulin G (1:200, ZSGB-BIO, China). Finally, the nucleus was stained with DAPI before the slides were mounted. Sections were then analyzed under a wide-field fluorescence microscope (Leica, Wetzlar, Germany) with 400× magnification. Fields of view were randomly selected by an observer blinded to experimental groups and counted by Image-Pro Plus software (Media Cybernetics, USA).

### Statistical analysis

Quantitative data were presented as the mean ± S.E.M. The statistical comparisons were performed by Student’s *t*-test for two groups or one-way ANOVA followed by Tukey’s test for at least three groups. *P* < 0.05 was considered statistically significant.

## Electronic supplementary material

Supplementary Tab.1

Supplementary Tab.2

Supplementary Fig.1

Supplementary Fig.2

Supplementary Fig.3

Supplementary Fig.4

Supplementary Fig.5
