# Supplementary material for: The autophagic degradation of Cav-1 contributes to PA-induced apoptosis and inflammation of astrocytes
Source: Cell Death Dis. 2018 Jul 10;9(7):771. doi: 10.1038/s41419-018-0795-3 (PMC6039485; doi:10.1038/s41419-018-0795-3)
Supplement: Supplementary file 1 — Supplementary Tab.1 [file 41419_2018_795_MOESM1_ESM.docx]

Tab.1 Oligonucleotide primers for real-time qPCR

| mRNA target Sense/anti-sense | |
| --- | --- |
| Cav-1 | 5’-TCTACAAGCCCAACAACAAGGCC-3’ |
|  | 5’-TGCACTGAATCTCAATCAGGAAGC-3’ |
| IL-1β | 5’- GGAACCCGTGTCTTCCTAAAG-3’ |
|  | 5’- CTGACTTGGCAGAGGACAAAG-3’ |
| TNF-α | 5’- CCACCACGCTCTTCTGTCTA-3’ |
|  | 5’- TGATCTGAGTGTGAGGGTCTG-3’ |
| IL-6 | 5’-ACAGTGCATCATCGCTGTTC-3’ |
|  | 5’-CCGGAGAGGAGACTTCACAG -3’ |
| ATG5 | 5’-TGAAGGAAGTTGTCTGGATAGCTCA-3’ |
|  | 5’-AAGTCTGTCCTTCCGCAGTC-3’ |
| ATG7 | 5’- ACCCTGCACAACACCAACAC-3’ |
|  | 5’- GAGCATGGGGTTTTCGAGAG-3’ |
| β-actin | 5’-CAACGAGCGGTTCCGAT-3’ |
|  | 5’-GCCACAGGATTCCATACCCA-3’ |
