# Supplementary material for: The autophagic degradation of Cav-1 contributes to PA-induced apoptosis and inflammation of astrocytes
Source: Cell Death Dis. 2018 Jul 10;9(7):771. doi: 10.1038/s41419-018-0795-3 (PMC6039485; doi:10.1038/s41419-018-0795-3)
Supplement: Supplementary file 2 — Supplementary Tab.2 [file 41419_2018_795_MOESM2_ESM.docx]

| Physiological index | Con | HFD |
| --- | --- | --- |
| Weight (gm) | 624.7±23.2 | 726.2±34.4** |
| serum total cholesterol (mmol/l) | 1.67±0.32 | 1.85±0.21 |
| serum triglyceride (mmol/l) | 1.04±0.35 | 2.41±0.43** |
| serum FFA (mmol/l) | 0.97±0.17 | 1.618±0.32** |

Tab.2 body weight and serum parameters

*FFA, free fat acid. Data are the means* ± *S.E.M. **P<0.01vs control group.*
