# Supplementary figures and images for: The autophagic degradation of Cav-1 contributes to PA-induced apoptosis and inflammation of astrocytes
Source: Cell Death Dis. 2018 Jul 10;9(7):771. doi: 10.1038/s41419-018-0795-3 (PMC6039485; doi:10.1038/s41419-018-0795-3)

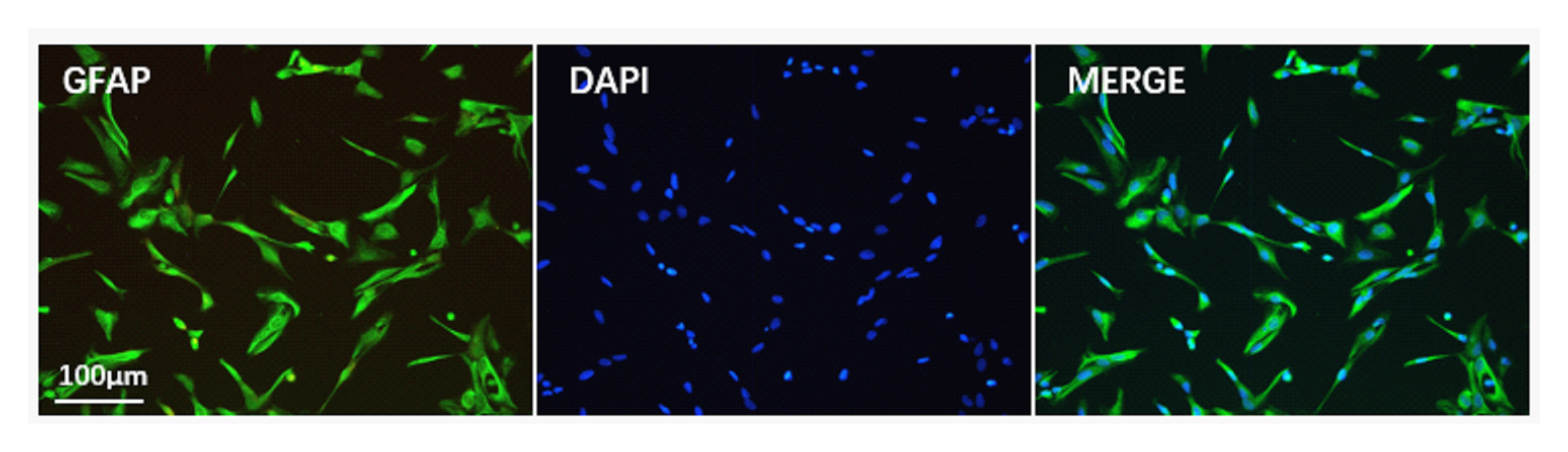

Supplement: Supplementary file 3 — Supplementary Fig.1 [file 41419_2018_795_MOESM3_ESM.tif]

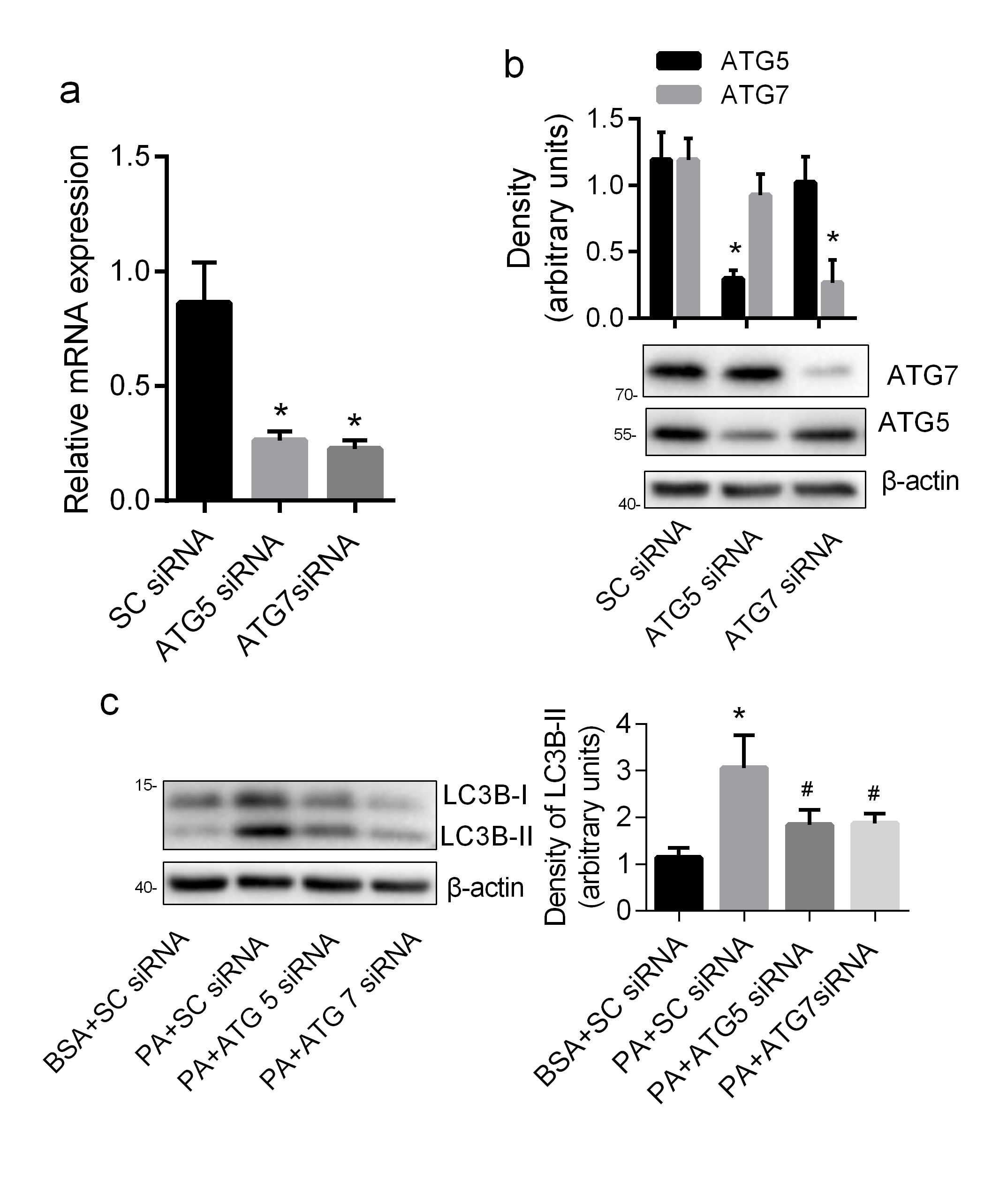

Supplement: Supplementary file 4 — Supplementary Fig.2 [file 41419_2018_795_MOESM4_ESM.tif]

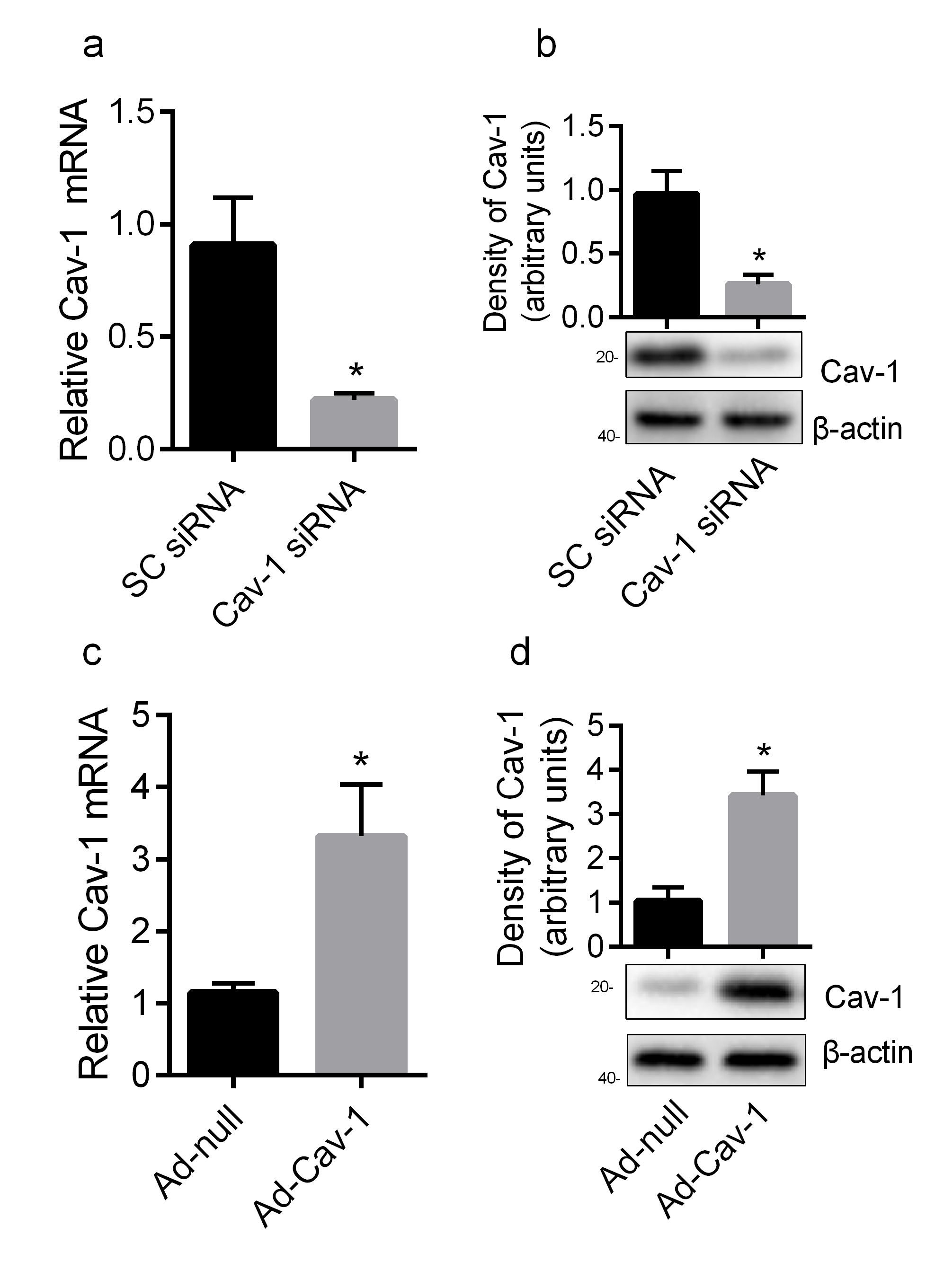

Supplement: Supplementary file 5 — Supplementary Fig.3 [file 41419_2018_795_MOESM5_ESM.tif]

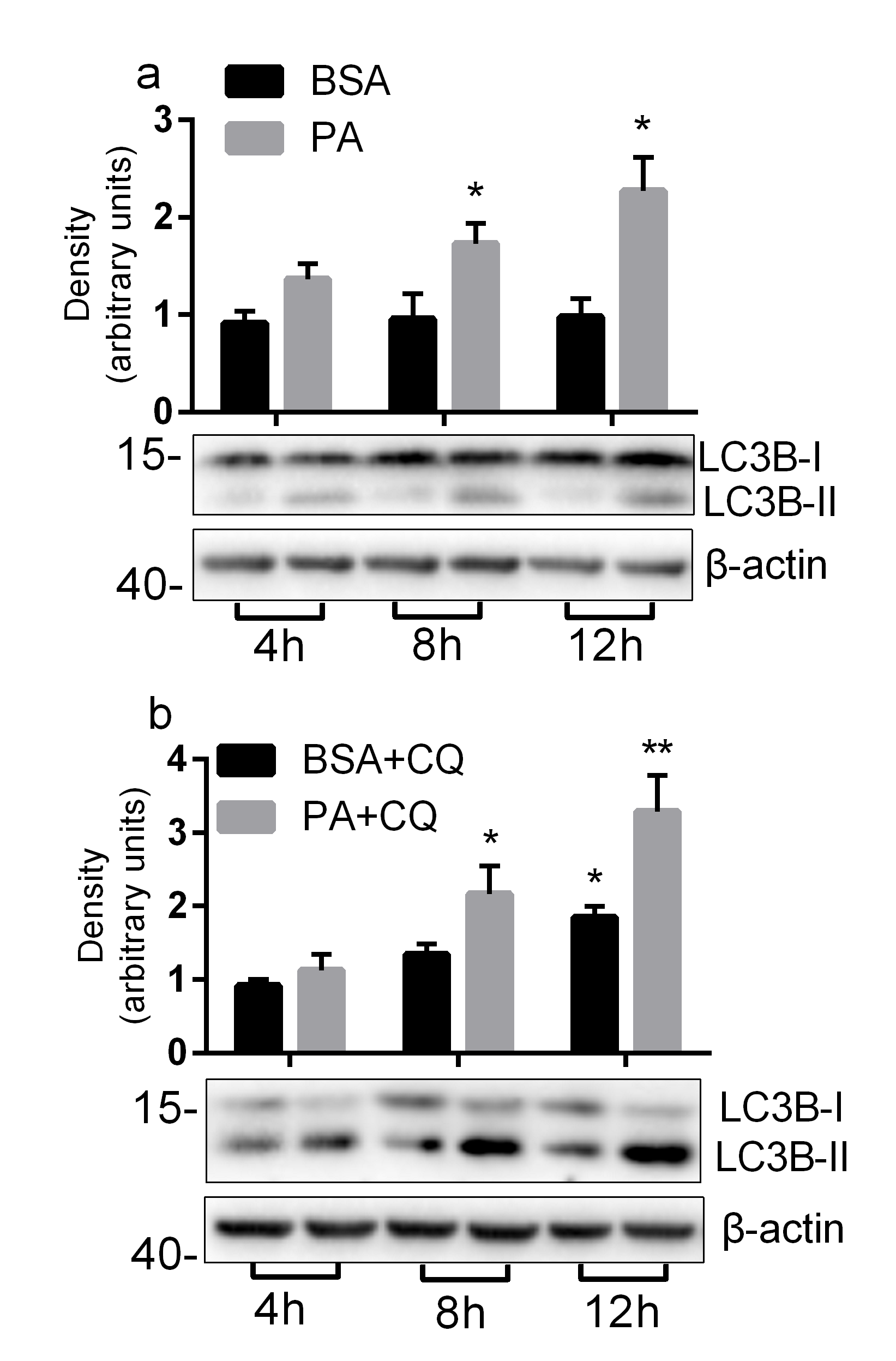

Supplement: Supplementary file 6 — Supplementary Fig.4 [file 41419_2018_795_MOESM6_ESM.tif]

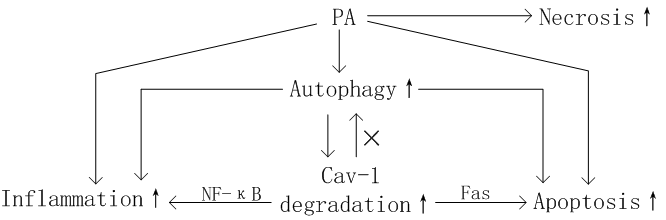

Supplement: Supplementary file 7 — Supplementary Fig.5 [file 41419_2018_795_MOESM7_ESM.tif]
